# Neighborhood walkability and cardiometabolic risk factors in australian adults: an observational study

**DOI:** 10.1186/1471-2458-13-755

**Published:** 2013-08-15

**Authors:** Falk Müller-Riemenschneider, Gavin Pereira, Karen Villanueva, Hayley Christian, Matthew Knuiman, Billie Giles-Corti, Fiona C Bull

**Affiliations:** 1 Saw Swee Hock School of Public Health and Department of Medicine, Yong Loo Lin School of Medicine, National University of Singapore, Block MD3, 16 Medical Drive, Singapore 117597, Singapore; 2 Institute for Social Medicine, Epidemiology and Health Economics - Charité University Medical Centre Berlin, Luisenstrasse 57, 10117 Berlin, Germany; 3 Yale School of Public Health, Center for Perinatal, Pediatric and Environmental Epidemiology, Yale University, 60 College Street, New Haven, CT 06520-8034, USA; 4 Centre for the Built Environment and Health, School of Population Health, The University of Western Australia, 35 Stirling Highway, Crawley, WA 6009, Australia; 5 School of Population Health, The University of Western Australia, 35 Stirling Highway, Crawley, WA 6009, Australia; 6 McCaughey Centre, VicHealth Centre for Community Welbeing, Melbourne School of Population and Global Health, University of Melbourne, Level 5, 207 Bouverie Street, Victoria 3010, Australia

**Keywords:** Built environment, Walkability, Cardiometabolic risk factors

## Abstract

**Background:**

Studies repeatedly highlight associations between the built environment and physical activity, particularly walking. Fewer studies have examined associations with cardiometabolic risk factors, with associations with obesity inconsistent and scarce evidence examining associations with other cardiometabolic risk factors. We aim to investigate the association between neighborhood walkability and the prevalence of obesity, hypertension, hypercholesterolaemia, and type-2 diabetes mellitus.

**Methods:**

Cross-sectional study of 5,970 adults in Western Australia. Walkability was measured objectively for a 1,600 m and 800 m neighborhood buffer. Logistic regression was used to assess associations overall and by sex, adjusting for socio-demographic factors. Mediation by physical activity and sedentary behavior was investigated.

**Results:**

Individuals living in high compared with less walkable areas were less likely to be obese (1,600 m OR: 0.84, 95% CI: 0.7 to 1; 800 m OR: 0.75, 95% CI: 0.62 to 0.9) and had lower odds of type-2 diabetes mellitus at the 800 m buffer (800 m OR: 0.69, 95% CI: 0.51 to 0.93). There was little evidence for an association between walkability and hypertension or hypercholesterolaemia. The only significant evidence of any difference in the associations in men and women was a stronger association with type-2 diabetes mellitus at the 800 m buffer in men. Associations with obesity and diabetes attenuated when additionally adjusting for physical activity and sedentary behavior but the overall association with obesity remained significant at the 800 m buffer (800 m OR: 0.78, 95% CI: 0.64 to 0.96).

**Conclusions:**

A protective association between neighborhood walkability and obesity was observed. Neighborhood walkability may also be protective of type-2 diabetes mellitus, particularly in men. No association with hypertension or hypercholesterolaemia was found. This warrants further investigation. Findings contribute towards the accumulating evidence that city planning and policy related strategies aimed at creating supportive environments could play an important role in the prevention of chronic diseases.

## Background

Chronic diseases such as cardiovascular disease (CVD) are the leading cause of death in most industrialized and many lower income countries [1,2]. It is well established that common modifiable risk factors, such as physical inactivity, unhealthy diet, obesity, hypertension and type-2 diabetes mellitus are responsible for the majority of cardiovascular and chronic disease events [1,3]. However, the prevalence of these risk factors has increased in many populations in recent years [4-10]. This raises concerns about the global impact on health systems if non-communicable disease rates continue to increase [1].

To counter the increase in CVD risk factors, environmental interventions have become increasingly recognized as necessary and potentially efficacious approaches to support behavior change [11-13]. Characteristics of the built environment include types of land use, population and housing density, connectivity of the street network, destinations accessed in daily life for shopping, education, work, and recreation, as well as the presence and quality of parks and public open spaces [14]. A substantial amount of research has linked many of these built environmental factors or composite measures of the built environment, such as walkability (residential density, street connectivity and mix of land uses), to physical activity behavior, including walking, cycling, active transportation or recreational physical activity [14-19]. Overall, adults in more supportive built environments, for example, pedestrian friendly areas with well-connected streets, higher density and a mix of land uses, are more likely to meet physical activity recommendations [17].

In addition, characteristics of the built environment may also have an important influence on cardiometabolic risk factors (CMRF), such as obesity, hypertension, hypercholesterolaemia, and type-2 diabetes mellitus, either directly or mediated through their influence on physical activity. In a recent systematic review of 131 studies of the association between environmental factors and CMRF, a substantial number of studies focused on measures of body weight. Findings indicate that features of the built environment (e.g., residential density, degree of urbanization, street intersections) are associated favorably with body weight, body-mass index (BMI) or obesity prevalence [20]. However, other reviews have concluded that these associations are inconsistent [14,15]. Moreover, other CMRF, including hypertension, hypercholesterolaemia, type-2 diabetes mellitus and the metabolic syndrome have received considerably less attention. Few studies have investigated the association between blood pressure and built environment features such as walkability, population density or greenness [20]. Nonetheless, the limited evidence suggests that supportive built environments are associated with favorable blood pressure levels or lower prevalence of hypertension [21-23]. Only two studies have investigated the influence of neighborhood resources or neighborhood housing conditions on type-2 diabetes mellitus [24,25], and there appears to be no studies examining associations between the built environment and hypercholesterolaemia or the metabolic syndrome [20].

The aim of this study was to complement the existing evidence by investigating the association between objectively measured walkability of the neighborhood and the prevalence of CMRF (obesity, hypertension, hypercholesterolaemia, and type-2 diabetes mellitus) in a large representative sample of adults in Perth, Western Australia.

## Methods

### Study design and study population

This was a cross-sectional study of adults (25+ years) [26,27]. Participants were included in the present study if they were residents of the Perth metropolitan area, participated in the Western Australian Health and Wellbeing Surveillance System (HWSS) survey between 2003 and 2006, and had consented to data linkage. The HWSS is a computer-assisted telephone interview based on a stratified random population sample administered by the Department of Health of Western Australia [28]. Those who consented to having their data linked to other health-related datasets were included in the study (5,970; 73% of 8,178 who completed the survey between 2003 and 2006). The sample’s baseline characteristics differed slightly from the non-linked population with the linked sample being somewhat older and with a higher prevalence of obesity, hypertension, hypercholesterolaemia, and type-2 diabetes mellitus.

Ethics approval was obtained from the Human Research Ethics Committee of the Department of Health of Western Australia and The University of Western Australia (#2010/1).

### Outcomes

Self-report of prior medically diagnosed or treated CMRF was obtained from the HWSS survey. The outcomes investigated in this study were obesity, hypertension, hypercholesterolaemia, and type-2 diabetes mellitus. Obesity was assessed based on self-reported height and weight and defined as a BMI ≥ 30 kg/m^2^. Participants were considered to have hypertension, hypercholesterolaemia or type-2 diabetes mellitus if they were previously diagnosed by a doctor, or if they were currently treated with medication for any of these conditions.

### Walkability

The built environment and its association with physical activity and other health related outcomes has been investigated using various methodological approaches, with different strengths and weaknesses [29]. In our study the built environment was assessed objectively using Geographical Information Systems (GIS) software (ESRI ArcGIS v10). Similar to many previous studies we calculated a walkability index to investigate the association between the built environment and CMRF [30-33]. This offers opportunities for comparison with the existing literature and to put our findings into perspective. The methodology of our study has been described in detail elsewhere [26]. Briefly, we combined three environmental measures: 1) residential density (ratio of number of residential dwellings to residential area in hectares) [31]; 2) street connectivity (ratio of count of three or more way intersections to area in km^2^) [31]; and 3) land use mix (calculated using an entropy formula (28) adapted from that originally used by Frank et al. (2005), which incorporates the proportion of area covered by each land use type by the summed area for all land use types of interest divided by the number of land use classes) [31,33]. These measures were calculated within two road network neighborhood buffers (i.e. service areas). A neighborhood buffer of 1,600 m was used because this distance represents approximately how far a participant can walk from home at a moderate to vigorous intensity pace, within 15 minutes [34]. Other studies have used smaller buffer sizes to characterize the neighborhood environment [29]. For that reason we additionally calculated walkability at a neighborhood buffer of 800 m reflecting the more immediate neighborhood environment. The walkability index was calculated by summing the z-scores for each component. Previous studies have frequently divided walkability scores into quartiles or quintiles to investigate associations with relevant outcomes as compared to investigations of linear associations with continuous measures [23,31,33]. In this study we have compared participants living in areas with high walkability (highest quartile) with those living in less walkable neighborhoods (other three quartiles). This was done to facilitate comparison of approach and results with other studies and also because Perth and other Australian cities do not have large variation in walkability and selecting out the top 25% preserves an adequate number for maintenance of statistical power and provides opportunity to determine what is achievable if the walkability of all environments were increased to this level. The classification of walkability (high and less walkable, respectively) at 1,600 m and 800 m was the same in 4,797 (80%) participants.

### Statistical analysis

Analyses were conducted for the total sample, and stratified by sex. Descriptive analyses of participant characteristics and the prevalence of CMRF are presented as percentages. Differences in the prevalence of CMRF between people living in high compared with low walkable neighborhoods were investigated using logistic regression models. Four binary outcomes were analyzed for the presence of each of the four CMRF separately. Models were fitted to estimate the odds ratio comparing odds of the risk factor (in the high vs. less walkable neighborhoods). Models adjusted for sex, age, education level, marital status and household income (Model A), with additional adjustment for physical activity and sedentary behavior (Model B). The impact of daily fruit and vegetable intake was explored in the context of Model B. These additional adjustments had little impact on observed associations and results are not presented separately. Comparison of the strengths of association in men vs. women was conducted using walkability sex interaction terms. Levels of physical activity were assessed by self-report. Time spent in vigorous and moderate activities was combined. Sedentary behavior was also assessed by self-report and reflects the time spent watching TV, playing video games or using the computer. All analyses were conducted separately for walkability at a neighborhood buffer of 1,600 m and 800 m.

## Results

### Study population and prevalence of cardiometabolic risk factors

Overall, 2,509 participants were male (42%) and 3,461 were female (58%) (Table 1). Hypercholesterolaemia was the most prevalent CMRF (35%), followed by hypertension (32.6%), obesity (18.8%) and type-2 diabetes mellitus (7%). CMRF prevalence was similar in men and women. Nearly one quarter (23.8%) of participants reported two or more CMRF.

**Table 1:** **Sociodemographic characteristics**, **physical activity and cardiometabolic risk factors overall and separately for men and women**

	**Total**		**Male**		**Female**	
	**N**	**%**	**N**	**%**	**N**	**%**
**Participants**	5970	100	2509	42.0	3461	58.0
**Age group**						
**25-44 years**	1783	29.9	700	27.9	1083	31.3
**45-64 years**	2432	40.7	1002	39.9	1430	41.3
**65+ years**	1755	29.4	807	32.2	948	27.4
**Educational level**						
**Less than year 10**	488	8.8	179	7.8	309	9.6
**Year 10 or year 11**	1325	23.9	421	18.2	904	28.0
**Year 12**	677	12.2	247	10.7	430	13.3
**Tafe/Trade qualification**	1726	31.2	860	37.3	866	26.8
**Tertiary degree or equivalent**	1321	23.9	601	26.0	720	22.3
**Marital status**						
**Married**	3797	63.6	1641	65.5	2156	62.3
**Living with a partner**	368	6.2	157	6.3	211	6.1
**Widowed**	571	9.6	127	5.1	444	12.8
**Divorced**	505	8.5	207	8.3	298	8.6
**Separated**	188	3.2	77	3.1	111	3.2
**Never married**	537	9.0	298	11.9	239	6.9
**Household income > 40,000 Australian $**	2971	54.2	1318	55.9	1653	53.0
**Walkability 1600m neighborhood buffer**						
**Less**	4528	75.8	1910	76.1	2618	75.6
**High**	1442	24.2	599	23.9	843	24.4
**Walkability 800m neighborhood buffer**						
**Less**	4595	77.0	1914	76.3	2681	77.5
**High**	1375	23.0	595	23.7	780	22.5
**Physical activity (≥ 150 minutes/week)**	1376	32.7	702	41.3	674	26.8
**Obesity (BMI ≥ 30 kg/m**^**2**^**)**	1074	18.8	422	17.2	652	20.0
**Hypertension**	1890	32.6	773	31.8	1117	33.2
**Hypercholesterolaemia**	1685	35.0	741	35.4	944	34.6
**Type-2 diabetes mellitus**	417	7.0	226	9.0	191	5.5
**Two or more cardiometabolic risk factors**	1419	23.8	618	24.6	801	23.1

### Association between walkability and CMRF

The observed prevalence of obesity, hypertension and type-2 diabetes was lower in high compared with less walkable areas at both the 1,600 m and 800 m neighborhood buffer (Tables 2 and 3). In contrast, the prevalence of hypercholesterolaemia was higher in more walkable areas (1,600 m and 800 m neighborhood buffers).

**Table 2:** **Prevalence and adjusted odds ratios of cardiometabolic risk factors for participants of high vs**. **less walkable neighborhoods** (**at 1**,**600 m buffer**) **overall**, **and for men and for women separately**

			**Model A**			**Model B**		
	**Less walkable neighborhoods (%)**	**High walkable neighborhoods (%)**	**OR**	**95% CI**	**p-value**	**OR**	**95% CI**	**p-value**
		**overall**						
**Obesity (BMI ≥ 30 kg/m2)**	19.7	15.8	**0**.**84**	**0**.**7 to 1**	**0**.**049**	0.86	0.7 to 1.05	0.139
**Hypertension**	33.0	31.3	0.93	0.8 to 1.08	0.336	0.91	0.75 to 1.09	0.299
**Hypercholesterolaemia**	34.7	35.6	1.02	0.88 to 1.2	0.771	1.04	0.85 to 1.26	0.711
**Diabetes mellitus**	7.2	6.5	0.95	0.72 to 1.25	0.726	1.08	0.72 to 1.62	0.701
		**male**						
**Obesity (BMI ≥ 30 kg/m2)**	18.2	14.3	0.75	0.56 to 1.01	0.056	0.82	0.59 to 1.14	0.229
**Hypertension**	32.3	30.2	0.9	0.72 to 1.14	0.398	1	0.74 to 1.34	0.981
**Hypercholesterolaemia**	35.3	35.6	1.05	0.83 to 1.32	0.687	1.1	0.82 to 1.48	0.526
**Diabetes mellitus**	9.1	8.9	1.14	0.78 to 1.66	0.489	1.26	0.72 to 2.21	0.425
		**female**						
**Obesity (BMI ≥ 30 kg/m2)**	20.9	16.9	0.89	0.71 to 1.12	0.319	0.88	0.68 to 1.14	0.336
**Hypertension**	33.5	32.1	0.94	0.77 to 1.14	0.53	0.86	0.67 to 1.09	0.203
**Hypercholesterolaemia**	34.3	35.7	1	0.81 to 1.23	0.994	1	0.77 to 1.3	0.991
**Diabetes mellitus**	5.8	4.8	0.76	0.51 to 1.15	0.194	0.92	0.51 to 1.66	0.779

BMI: Body Mass Index, OR: odds ratio, CI: confidence interval, Model A: Less walkable neighborhoods=reference category; Odds ratios adjusted for age, sex, education level, household income, and marital status, Model B: Less walkable neighborhoods=reference category; Odds ratios adjusted for age, sex, education level, household income, marital status, plus physical activity and sedentary behavior, Bold data: highlight statistically significant associations (p-value <0.05).

**Table 3:** **Prevalence and adjusted odds ratios of cardiometabolic risk factors for participants of high vs**. **less walkable neighborhoods** (**at 800 m buffer**) **overall**, **and for men and for women separately**

			**Model A**			**Model B**		
	**Less walkabie neighborhoods (%)**	**High walkabie neighborhoods (%)**	**OR**	**95% CI**	***p*-value**	**OR**	**95% CI**	***p*-value**
**overall**								
**Obesity (BMI ≥ 30 kg/m2)**	20.9	16.9	**0**.**75**	**0**.**62 to 0**.**9**	**0**.**002**	**0**.**78**	**0**.**64 to 0**.**96**	**0**.**018**
**Hypertension**	33.5	32.1	0.93	0.8 to 1.08	0.33	0.97	0.81 to 1.16	0.743
**Hypercholesterolaemia**	34.3	35.7	1.09	0.93 to 1.28	0.267	1.11	0.91 to 1.34	0.304
**Diabetes mellitus**	5.8	4.8	**0**.**69**	**0**.**51 to 0**.**93**	**0**.**016**	0.79	0.52 to 1.21	0.282
**male**								
**Obesity (BMI ≥ 30 kg/m2)**	18.2	14.1	**0**.**73**	**0**.**55 to 0**.**98**	**0**.**035**	0.76	0.55 to 1.04	0.089
**Hypertension**	32.6	29.2	0.89	0.71 to 1.13	0.348	0.94	0.71 to 1.26	0.694
**Hypercholesterolaemia**	34.9	37.0	1.17	0.93 to 1.48	0.174	1.14	0.85 to 1.52	0.375
**Diabetes mellitus**	10.2	5.4	**0**.**53**	**0**.**34 to 0**.**82**	**0**.**005**	0.6	0.32 to 1.14	0.122
**female**								
**Obesity (BMI ≥ 30 kg/m**^**2**^**)**	20.9	16.7	**0**.**76**	**0**.**6 to 0**.**96**	**0**.**02**	0.8	0.61 to 1.04	0.093
**Hypertension**	33.3	32.8	0.95	0.78 to 1.16	0.604	0.99	0.78 to 1.25	0.913
**Hypercholesterolaemia**	34.1	36.5	1.02	0.82 to 1.26	0.857	1.08	0.84 to 1.4	0.551
**Diabetes mellitus**	5.6	5.1	0.88	0.58 to 1.32	0.53	0.97	0.54 to 1.73	0.917

BMI: Body Mass Index, OR: odds ratio, CI: confidence interval, Model A: Less walkable neighborhoods=reference category; Odds ratios adjusted for age, sex, education level, household income, and marital status Model B: Less walkable neighborhoods=reference category; Odds ratios adjusted for age, sex, education level, household income, marital status, plus physical activity and sedentary behavior, Bold data: highlight statistically significant associations (p-value <0.05).

After adjusting for socio-demographic factors (Tables 2 and 3, model A), individuals living in high compared with less walkable areas were not as likely to be obese (1,600 m OR: 0.84, 95% CI: 0.7 to 1; 800 m OR: 0.75, 95% CI: 0.62 to 0.9). Individuals in high walkable neighborhoods also had lower odds of type-2 diabetes mellitus. However, this relationship was only statistically significant at the 800 m neighborhood buffer (1,600 m OR: 0.95, 95% CI: 0.72 to 1.25; 800 m OR: 0.69, 95% CI: 0.51 to 0.93). There was little evidence of an association between walkability and hypertension or hypercholesterolaemia.

Whilst the estimated association between walkability and obesity was stronger in men than women (Tables 2 and 3) the difference did not reach statistical significance (1,600 m OR: 0.75 for men vs. 0.89 for women, p-value=0.414; 800 m OR: 0.73 for men vs. 0.76 for women, p-value=0.898). The estimated association between walkability and type-2 diabetes mellitus was stronger in men than women at the 800 m neighborhood buffer (OR: 0.53 for men vs. 0.88 for women, p-value=0.083) but not at the 1,600 m buffer (OR 1.14 for men vs. 0.76 for women, p-value=0.204). No significant relationships between walkability and hypertension and hypercholesterolaemia were found for either men or women.

The mediation effect of physical activity and sedentary behavior on the relationship between walkability and CMRF (Table 2 and 3, model B) was investigated. After adjusting for these factors, previously statistically significant associations between walkability and obesity attenuated overall (1,600 m OR: 0.84 to 0.86; 800 m OR: 0.75 to 0.78), in men (1,600 m OR: 0.75 to 0.82; 800 m OR: 0.73 to 0.76) and in women (800 m OR: 0.76 to 0.80) and only the association with obesity overall at the 800 m neighborhood buffer remained significant (1,600 m p-value=0.139; 800 m p-value=0.018). Similarly, the significant associations between walkability and type-2 diabetes mellitus at the 800 m buffer attenuated overall, in men and in women and were no longer statistically significant.

## Discussion

In this large representative sample of adults we observed a substantial risk factor burden indicating that almost one quarter of the population are highly susceptible to cardiometabolic morbidity.

Our findings showed strong and consistent negative associations between the objectively measured walkability of the immediate (800 m) and extended (1,600 m) residential neighborhood and the prevalence of obesity. For type-2 diabetes mellitus, strong negative associations were also observed at the 800 m neighborhood buffer and this was only significant in men. Furthermore, no consistent associations between walkability and hypertension and hypercholesterolaemia were observed.

A number of studies have investigated the associations between characteristics of the built environment and obesity [20]. However, the majority of this research has been conducted in the United States and overall there have been mixed findings [20,30,35,36]. In contrast to our findings, a recent study from the same region that also used a walkability index to measure the built environment (Western Australia) found no association between walkability and body mass index [37]. This might be explained by differences in the study population (the current study consists of a large population representative sample vs. the previous study consisted of home owners who moved into new housing developments). Moreover, differences in the outcome classification might also explain this apparently contradictory finding.

Overall, associations between neighborhood walkability and CMRF appeared to be stronger and more consistent in the immediate (800 m) compared with the extended (1,600 m) neighborhood, as well as in men compared with women. The observed sex difference may, in part, be due to a more walkable or more pedestrian friendly environment having a larger influence on the physical activity behavior and in turn weight status and risk of type-2 diabetes mellitus in men than women. Mediation analyses provided some confirmation of this as the association between walkability and obesity was partly explained by physical activity and sedentary behavior. However, a considerable amount of the association between walkability and obesity was not explained by physical activity and sedentary behavior in our study. One possible explanation for this observation is, that a more context-specific measure of physical activity, such as neighborhood level physical activity (vs. a general measure of total physical activity as used in our study) may more adequately and, to a greater extent, explain the association between neighborhood walkability and obesity [38]. Similarly, a measure of sitting time at home as compared to total sitting time, which is largely affected by the work environment, for the measurement of sedentary behavior may explain observed associations. Previous research that reported the proportion of physical activity within and outside the residential neighborhood offers some support to this hypothesis [34]. Also, objectively and thus more accurately measured physical activity and sedentary behavior may be able to explain observed associations more adequately. However, this study was restricted to using a self-report measure of total physical activity and sedentary behavior as captured by the HWSS survey. The fact that neighborhood walkability was less strongly associated with obesity and type-2 diabetes mellitus in women raises the question as to whether the influence of specific built environment factors differs by sex. For instance, other factors of the built environment such as the availability and accessibility of neighborhood destinations (e.g., food outlets, parks and recreation venues) and perceived and real safety may have a greater influence on obesity and other CMRF in women than in men [39,40]. Future research should explore the relationship between other built environment factors and CMRF separately for men and women and consider using neighborhood level measures of physical activity. Moreover, the more proximate neighborhood appears to be more important for cardiometabolic outcomes and this should be further investigated in future studies.

Our findings with regard to type-2 diabetes mellitus, hypertension, and hypercholesterolaemia are only partly consistent with previous research. To date, the majority of studies have investigated the association between environmental factors such as noise exposure and hypertension or dyslipidaemia [20]. Among the small number of published studies investigating the influence of the built environment only one used a walkability index [21-23,41]. Generally, however, studies found that a supportive built environment (e.g. high walkability, high population density) was positively associated with blood pressure levels or hypertension prevalence [21-23,41]. In contrast, we found no significant relationship between walkability and hypertension. A recent systematic review identified only two studies investigating the association between the built environment and type-2 diabetes mellitus but none of these studies measured walkability. Studies found that housing conditions and more resourceful neighborhoods (suitability of the environment for physical activity and availability of healthy foods) was associated with a lower incidence of type-2 diabetes mellitus [24,25]. In support of prior findings, our study found that walkability was negatively associated with type-2 diabetes mellitus in men only and at the 800m neighborhood buffer. This finding is plausible and somewhat compatible with our findings that particularly in men the immediate neighborhood (800 m buffer) was more strongly and more consistently associated with obesity. Body weight and obesity, respectively, are among the strongest risk factors for type-2 diabetes mellitus [42-44]. Hypothetically, the stronger association between the walkability and obesity in men and in the immediate neighborhood could explain the observed association between walkability and type-2 diabetes mellitus. These findings warrant further investigation and confirmation in future studies. Finally, our study appears to be the first to investigate the association between the built environment and hypercholesterolaemia [20], however no significant association was observed.

A number of factors may explain the variation between our findings and that of previous research. For instance, our study had a representative sample of the adult population aged 25 and older. Many previous studies used a different methodology and were not based on representative adult population samples but specifically targeted certain population groups, such as middle aged people [22,23] or populations with specific ethnic origins [21]. Furthermore, as previously indicated, we used walkability as a composite measure of the built environment whereas many other studies have focused on single environmental characteristics (e.g. neighborhood conditions, population density) [22,24]. Another potential factor could be the choice of neighborhood buffers used. We investigated the immediate and more extended neighborhood (800 m and 1,600 m neighborhood buffer) with associations being somewhat stronger at the 800 m neighborhood buffer. However, in contrast to our findings other studies have also reported associations between the built environment and type-2 diabetes mellitus and hypertension at a neighborhood buffer of 1,600 m [25,41].

A major strength of the current study is that it investigated multiple CMRF within the same study population. In addition, our study was based on a representative population sample with a high proportion of participants granting permission for data linkage. Furthermore, walkability was measured using objective attributes of the built environment. Nevertheless, when interpreting our findings a number of limitations should be considered. First, despite their frequent use, limitations of composite measures of the built environment (walkability index) have been highlighted [45][29]. Among others, these include the inability to identify specific relevant environmental components, the use of entropy scores that can be similar for different walking environments, and the fact that a wide range of land uses is usually not captured in existing walkability indices. On the other hand, using a walkability index could have the advantage of providing better estimates compared to individual and frequently inter-related environmental components [31]. In addition, focusing on a number of relevant environmental variables is likely necessary and could have greater potential to improve pedestrian friendliness of a neighborhood [31], Second, our study used self-reported outcome measures which could introduce measurement error. Future studies investigating multiple CMRF should include objective measures of CMRF’s. Third, although we used a composite measure of the built environment other potentially important built environment or neighborhood attributes (e.g., presence of food outlet destinations) were not considered and may differ by sex (e.g., neighborhood safety and crime). Fourth, this study had a cross-sectional design, which limits the ability to draw causal inferences. It should also be acknowledged that we were not able to identify factors related to the choice of residential neighborhood because the neighborhood and health data were linked by an independent government department. Adjustment for residential preference was therefore not possible within our study. However, we have longitudinal evidence that the influence of neighborhood selection might not be large [46]. Nevertheless, future studies should attempt to consider this aspect when investigating associations between the neighborhood environment and health related outcomes.

There is growing recognition that the global burden of preventable chronic disease is so great, that if uncurbed it could cripple health systems and undermine social and economic development [1]. Combating chronic diseases is therefore said to require a whole-of-society multi-sector approach, including consideration being given to city planning that support more active lifestyles such as daily walking and cycling [47]. While the associations observed in this study are relatively modest, small shifts across whole populations can have a large impact on the burden of disease. Thus, these results highlight the importance of engaging city planners to create walkable pedestrian-friendly neighborhoods as one key strategy in tackling key CMRF.

## Conclusion

In conclusion, we observed a strong protective association between neighborhood walkability and obesity. Neighborhood walkability may also be protective of type-2 diabetes mellitus, particularly in men. Associations were partly explained by the physical activity and sedentary behavior of participants. No consistent associations between walkability and hypertension or hypercholesterolaemia were found. Our findings provide further evidence of the influence of the built environment on health. City planning and policy-related strategies aimed at improving the connectivity of the street network, mix of land uses and density of housing would enable the necessary supportive environments for health-related behaviors and the prevention of chronic diseases. Future studies should examine other objectively measured attributes of the built environment, examine the relationship between features of the built environment and objectively assessed CMRF separately for men and women, and consider these relationships at other theoretically important neighborhood buffers.

## Competing interest

The authors declare that they have no competing interests.

## Authors’ contributions

FMR conducted the statistical analysis and drafted the manuscript. GP conceived the study and supported the statistical analysis. All authors were involved in redrafting of the manuscript, provided advice on re-analyses for successive drafts, and interpreted the results. All authors read and approved the final manuscript.

## Pre-publication history

The pre-publication history for this paper can be accessed here:

http://www.biomedcentral.com/1471-2458/13/755/prepub
